# Supplementary material for: Molecularly Targeted Therapies for Gastric Cancer. State of the Art
Source: Cancers (Basel). 2021 Aug 14;13(16):4094. doi: 10.3390/cancers13164094 (PMC8392056; doi:10.3390/cancers13164094)
Supplement: Supplementary file 1 [file cancers-13-04094-s001.zip › cancers-1301787-supplementary.pdf]

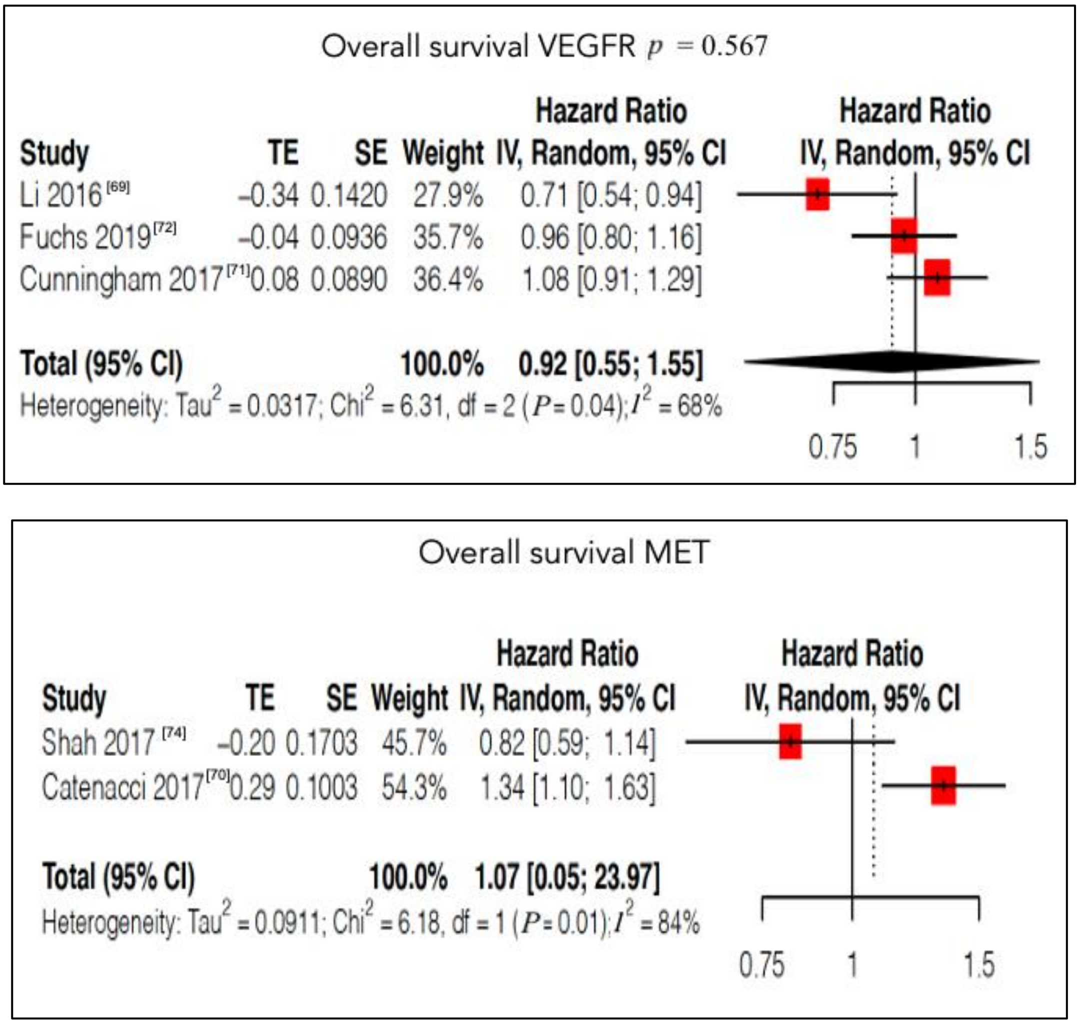

Figure S1. Forest plot of comparison: molecular-targeted therapy alone/plus chemotherapy versus chemotherapy alone/placebo. Main analyses; outcome: Overall Survival. Analysis of subgroups by type of molecular therapy (anti-VEGFR and anti-MET).

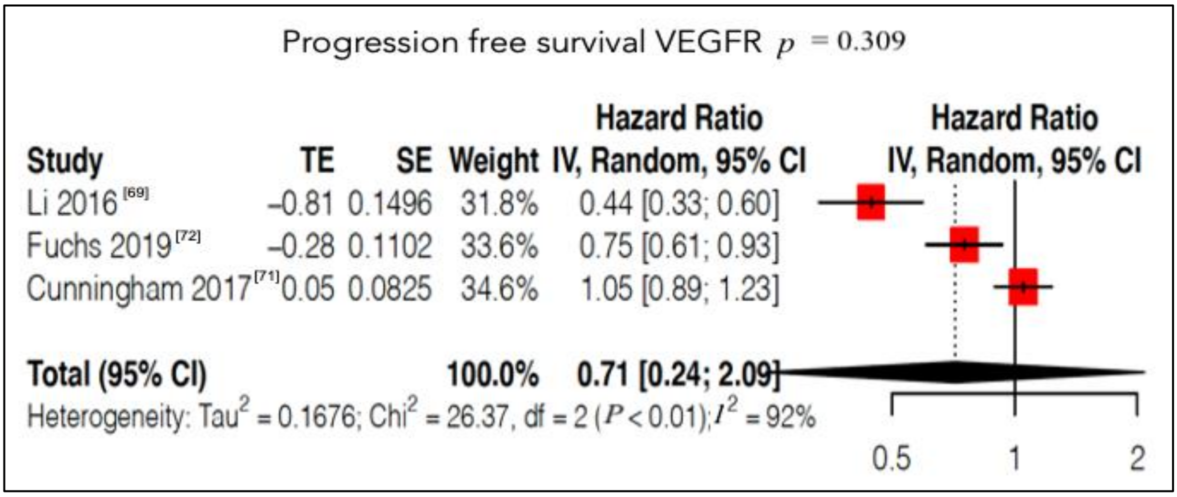

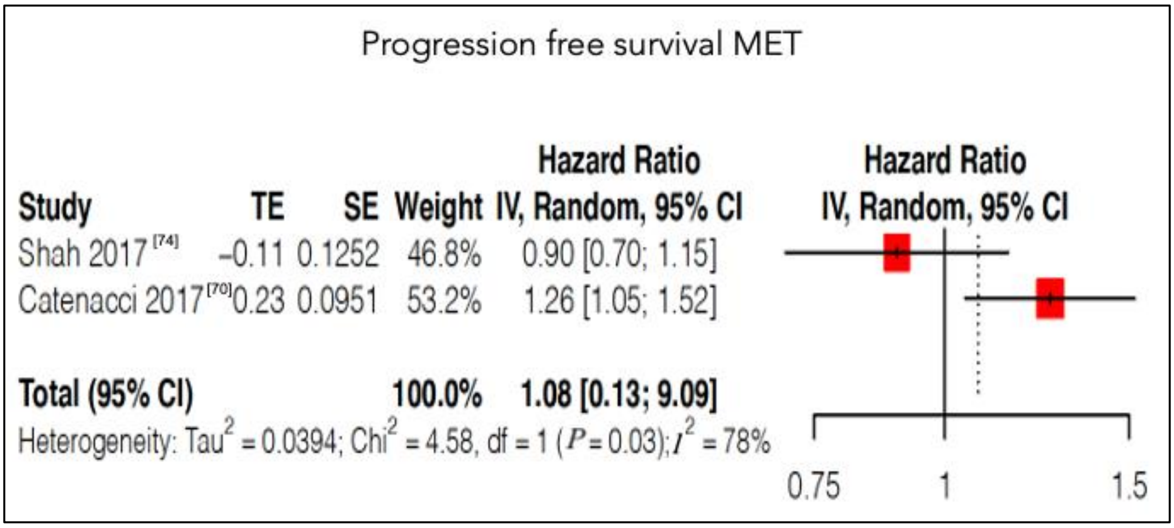

Figure S2. Forest plot of comparison: molecular-targeted therapy alone/plus chemotherapy versus chemotherapy alone/placebo. Main analyses; outcome: Progression Free Survival. Analysis of subgroups by type of molecular therapy (anti-VEGFR and anti-MET).

Table S1. Patient characteristics of studies included in the last Cochrane review.

Author, Year, Acronym	Nr Total pts (EXP/CTR)	Treatment Line (%)	Primary Endpoint	Setting	Molecular Target	EXP Arm	CTR Arm	Tumor Stage (%)	Tumor Site (%)
Bang [12], 2010 ToGA	594(294/290)	I II	OS	Adj	HER-2	Trastuzumab + fluoropyrimidine + cisplatin	Fluoropyrimidine + cisplatin	III IV	Stomach GEJ
Eatock {76}, 2013	171 (56 + 59/56)	I II	PFS	Adj	VEGF-A	Trebananib + CX	CX	IV	Stomach GEJ Distal oesophagus
Hecht [58], 2013	545 (272 /273)	I II	OS	Adj	HER-2	Lapatinib + XELOX	Placebo+ XELOX	III IV	Stomach GEJ Oesophagus
Iveson [77], 2014	121 (42 + 40/39)		OS	Adj	HGF	rilotumumab + ECX	Placebo + ECX	III IV	Stomach GEJ
Koizumi [78], 2013	93 (46 /47)	I	PFS	Adj	VEGFR-2	Orantinib + S-1/CDDP	S-1/CDDP	III	Stomach
Lordick [61], 2013 EXPAND	904 (455 / 449)	I II	PFS	Adj	EGFR	Cetuximab+ capecitabine and cisplatin	capecitabine and cisplatin	IV III	Stomach GEJ
Ohtsu [40], 2011 AVAGAST	774 (387 /387)	I	OS	Adj	VEGF	bevacizumab + capecitabine and cisplatin/FU	capecitabine and cisplatin/FU	III IV	Stomach GEJ
Rao [79], 2010	72 (36/36)	I	Objective response	Adj	EGFR	Matuzumab + ECX	ECX	IV	Stomach EGJ Distal oesophagus
Shen [80], 2015 AVATAR Study	202 (100 /102)	I	OS	Adj	VEGFR	Bevacizumab + CX	CX	III IV	Stomach GEJ
Waddell [55], 2013 REAL3	553(278/275)	I	OS	Adj	HER-1	Panitumumab + EOC	EOC	III IV	Stomach EGJ Oesophagus
Zhang [81], 2014	56 (30/26)	I II	OS	Adj	EGFR	Cetuximab + S-1 and oxaliplatin	S-1 and oxaliplatin	III IV	Stomach

Pts: patients; CX: cisplatin and capecitabine; XELOX: Capecitabine+ Oxaliplatin; ECX: epirubicin, cisplatin, and capecitabine; S-1: fluoropyrimidine; CDDP: cisplatin; EOC: epirubicin and oxaliplatin and capecitabine.
